# Prevalence, Attributes, and Risk Factors of QT-Interval-Prolonging Drugs and Potential Drug-Drug Interactions in Cancer Patients: A Prospective Study in a Tertiary Care Hospital

**DOI:** 10.7759/cureus.60492

**Published:** 2024-05-17

**Authors:** Akash Agnihotri, Saravana Kumar Ramasubbu, Arkapal Bandyopadhyay, Manjunath Bidarolli, Uttam Kumar Nath, Biswadeep Das

**Affiliations:** 1 Department of Pharmacology, Amrita School of Medicine, Faridabad, IND; 2 Department of Pharmacology, Andaman and Nicobar Islands Institute of Medical Sciences, Port Blair, IND; 3 Department of Pharmacology, All India Institute of Medical Sciences, Kalyani, Kalyani, IND; 4 Department of Pharmacology, All India Institute of Medical Sciences, Rishikesh, Rishikesh, IND; 5 Department of Medical Oncology and Hematology, All India Institute of Medical Sciences, Rishikesh, Rishikesh, IND

**Keywords:** prevalence study, pharmaco-epidemiology, risk factors for qt-prolongation, cancer patients, qt-interval prolonging drug-drug interactions

## Abstract

Introduction

Cancer chemotherapy regimens include multiple classes of adjuvant drugs as supportive therapy. Because of the concurrent intake of other drugs (like antiemetics, antidepressants, analgesics, and antimicrobials), there is a heightened risk for possible QT interval prolongation. There is a dearth of evidence in the literature regarding the usage of QT-prolonging anticancer drugs and associated risk factors that have the propensity to prolong QT interval. The purpose was to explore the extent of the use of QT-interval-prolonging drugs and potential QT-prolonging drug-drug interactions (QT-DDIs) in cancer patients attending OPD in a tertiary-care hospital.

Methods

This was a hospital-based, cross-sectional, observational study. Risk stratification of QT-prolonging drugs for torsades de pointes (TdP) was done by the Arizona Center for Education and Research on Therapeutics (AzCERT)/CredibleMeds-lists, and potential QT-DDIs were determined with four online DDI-checker-software.

Results

In 1331 cancer patients, the overall prevalence of potential QT-prolonging drug utilization was 97.3%. Ondansetron, pantoprazole, domperidone, and olanzapine were the most frequent QT-prolonging drugs in cancer patients. The top six antineoplastics with potential QT-prolonging and torsadogenic actions were capecitabine, oxaliplatin, imatinib, bortezomib, 5-fluorouracil, and bendamustine. Evidence-based pragmatic QTc interval prolongation risk assessment tools are imperative for cancer patients.

Conclusion

This study revealed a high prevalence of QT-prolonging drugs and QT-DDIs among cancer patients who are treated with anticancer and non-anticancer drugs. As a result, it's critical to take precautions, stay vigilant, and avoid QT-prolonging in clinical situations. Evidence-based pragmatic QTc interval prolongation risk assessment tools are needed for cancer patients.

## Introduction

Cancer remains the leading cause of both mortality and morbidity on a global scale, despite a consistent decline in the risk of cancer-related mortality. It's estimated that as of 2021, approximately four million cancer deaths have been averted [1]. QT interval prolongation is a well-established risk factor for fatal ventricular arrhythmias, alluded to as torsades de pointes (TdP), with consequent sudden cardiac death (SCD). In this aspect, cardiac arrhythmias can be life-threatening, and rising worry over cancer therapy-related cardiotoxicity has ushered in a new branch of medicine called cardio-oncology [2]. Congenital long QT syndrome, female sex, age over 65, structural heart disorders, and polypharmacy are all major QT prolongation risk factors. Furthermore, drug-drug interactions (DDIs) and electrolyte disbalance due to nausea and vomiting can elevate the risk of QT prolongation and TdP [3,4]. Drug-drug interactions frequently cause adverse drug reactions (ADRs). DDIs are responsible for between 3-26% of all ADRs that result in admission [5].

Patients with advanced cancer receive a diverse category of medications to address the cancer itself, alleviate associated signs and symptoms, manage concurrent disorders such as cardiomyopathy, diabetes, and dyslipidemia, and assist patients in coping with the adverse effects of chemotherapy. Polypharmacy does expose cancer patients to a high risk of hazardous drug-drug interactions (DDIs), which could be aggravated by impaired organ function [6].

Several novel anticancer drugs (histone deacetylase inhibitors and multitargeted tyrosine kinase inhibitors (TKIs), antipsychotics, and antihistaminics (e.g., terfenadine, cisapride, astemizole, and grepafloxacin) have been noted to delay cardiac repolarization and precipitate TdP occurrence in cancer patients. Many have been withdrawn from the market or are subject to restricted marketing or a black box warning [7,8]. An up-to-date list of drugs that can engender QT prolongation can be located online, and a recent addition was the drug imatinib under the “possible risk of TdP” category [9]. 

Furthermore, adjunctive drugs and supportive care can contribute to a variety of heart rhythm problems, the most notable of which is QT prolongation, which can lead to cardiac arrhythmias. The concurrent utilization of cancer drugs and palliative care may cause the QT interval to be prolonged.

Currently, pharmacoepidemiologic data regarding the extent of use of QT-prolonging anticancer drugs, potential QT-prolonging drug-drug interactions (QT-DDIs), and prevalence and characterization of risk factors related to the use of QT-prolonging drugs in cancer patients are scant. Therefore, this study was conducted to explore the medication use pattern, prevalence, and types of risk factors as well as estimate the potential QT-DDIs in connection with possible QT prolongation and TdP in cancer patients in the light of evidence gleaned from the scientific literature.

## Materials and methods

Study design and setting

This study was conducted as a prospective, hospital-based, cross-sectional study for a period of one year (from September 2020 to September 2021) at the medical-oncology and radiation-oncology departments of the All India Institute of Medical Sciences (AIIMS), Rishikesh, Uttarakhand, India.

Study population

Inclusion Criteria

All patients suffering from cancer (malignant/hematological malignancies), who attended cancer outpatient departments (OPDs), and who were receiving oral or intravenous treatment with one or more anticancer agents were included. This study encompassed all age groups and both genders.

Exclusion Criteria

Patients who were not willing to give written informed consent were excluded from this study. Research and ethical approval for this study were obtained from the Institutional Ethics Committee (Approval Letter No. AIIMS/IEC/20/610 dated September 12, 2020) of the All India Institute of Medical Sciences (AIIMS), Rishikesh, Uttarakhand, India.

Data collection procedure

In this study, the researchers (AA and BD) visited the medical oncology, hematology, and radiotherapy OPDs to collect prescriptions and treatment sheets of patients who attended the cancer OPDs from consecutive patients in a prospective manner. The survey was conducted for a total of one year.

All pertinent data in the context of patient characteristics like age, sex, location of residence (urban/rural), diagnosis, comorbidities, and information from the relevant prescriptions (i.e., the OPD treatment sheets and case record forms) were captured in a customized and specially designed proforma for this purpose (inclusive of socio-demographic and treatment sections).

Patients who received one or more ECGs during their initial and follow-up visits were recorded, in addition to individual risk factors for TdP, and all medications (antineoplastic or otherwise) taken that have been established to foster QT protraction.

Data analysis tools

Arizona Center for Education and Research on Therapeutics (AzCERT)/CredibleMeds List of QT-Prolonging Drugs

According to the most current version of the AzCERT/CredibleMeds QT drugs lists (dated January 2, 2023), we utilized the AzCERT/CredibleMeds list of QT-prolonging drugs as it is a widely recognized and reputable resource for identifying medications known to prolong the QT interval. QT-prolonging and TdP-provoking drugs were noted and risk-stratified into four groups [9]. The analysis excluded drugs that AzCERT/CredibleMeds has determined to have no bearing on QT protraction and TdP. Every drug in therapeutic use has been extensively appraised by the AzCERT/CredibleMeds team, and these evaluations are continually updated based on global clinical inputs and cues. The AzCERT/CredibleMeds Risk Stratification System categorizes QT-interval-prolonging torsadogenic medications into four lists: known risk of TdP (List-1), possible risk of TdP (List-2), conditional risk of TdP (List-3), and drugs to avoid in congenital long QT syndrome (CLQTS) (List-4) (Special risk (SR)).

Anatomic Therapeutic Chemical (ATC) Classification System Codes

The WHO Collaborating Centre for Drug Statistics Methodology's Anatomic Therapeutic Chemical (ATC) Classification System Codes have been utilized as appropriate [10].

Potential QT-Prolonging Drug-Drug Interactions (QT-DDIs) Analysis

Potential QT-DDIs were analyzed using four online drug-drug interactions checker software: “Drugs.com Drug-Drug Interactions Checker” [11], “Epocrates Multicheck Online Drug Interaction Checker” [12], “Medscape Drug Interaction Checker” [13], and “UpToDate’s Lexicomp® Drug Interactions” [14].

Statistical analysis

The socio-demographic information of patients, diagnoses, comorbidities, drug use patterns, and drug interaction characteristics have all been computed utilizing descriptive statistics. Categorical data have been reported as frequencies and percentages (%). Univariate and multivariate logistic regression analyses have been performed to determine the risk factors for the occurrence of potential QT-DDIs and to calculate the odds ratios (OR). A p-value of ≤ 0.05 was deemed statistically significant. For all statistical analyses, Microsoft Excel (Microsoft Corporation, Redmond, Washington, United States) and IBM SPSS Statistics for Windows, Version 23 (Released 2015; IBM Corp., Armonk, New York, United States). were utilized.

## Results

Among the 1331 patients in the study who visited the cancer outpatient departments (OPDs) during the designated study period, 640 (48.1%) were males. The mean age (± standard deviation (SD)) of the patients in this study was 48 ± 16 years. Around 470 (35.3%) were receiving ten or more drugs concurrently. The mean number (± SD) of prescribed drugs among patients was 8.8 ± 4.2. Breast cancer was the most commonly encountered cancer, accounting for 347 cases, or 26.1%. The most common comorbid illnesses were hypertension (76, 5.7%), diabetes mellitus (72, 5.4%), thyroid disorders (26, 1.9%), hepatitis C (17, 1.2%), and hepatitis B (8, 1.2%) (Table 1).

**Table 1:** Socio-demographic and clinical attributes of the cancer patients (n=1331) ^a^The percentage was determined using the total number of patients i.e., 1331 ^_b_^Overall prescribed drugs signify both QT-prolonging and non-QT-prolonging medications

Variable(s)	Patients n (%)^a^
Gender	
Male	640 (48.1)
Female	691 (51.9)
Age	
≤ 30	215 (16.2)
31-40	217 (16.3)
41-50	313 (23.5)
51-60	339 (25.5)
>60	247 (18.5)
Overall prescribed drugs^b^	
≤ 5	243 (18.2)
6-7	351 (26.4)
8-9	267 (20.1)
≥10	470 (35.3)
Diagnoses	
Breast cancer	347 (26.1)
Head and neck cancer	247 (18.6)
Gastrointestinal cancer	112 (8.4)
Lung cancer	96 (7.2)
Chronic myelogenous leukemia (CML)	81 (6.1)
Genitourinary cancer	60 (4.5)
Acute lymphoblastic leukemia (ALL)	58 (4.3)
Gynecological carcinoma	58 (4.3)
Ovarian cancer	49 (3.7)
Non-Hodgkin lymphoma	49 (3.7)
Hodgkin lymphoma	35 (2.7)
Colorectal carcinoma	33 (2.5)
Multiple myeloma	27 (2.0)
Musculoskeletal cancer	25 (1.9)
Brain cancer	17 (1.3)
Acute myelogenous leukemia (AML)	14 (1.0)
Chronic lymphocytic leukemia (CLL)	10 (0.7)
Skin	2 (0.1)
Carcinoma of unknown origin	2 (0.1)
Adenocarcinoma unknown	2 (0.1)
Essential thrombocytosis	1 (0.1)
Myelodysplastic syndrome (MDS)	1 (0.1)
Neuroendocrine carcinoma	1 (0.1)
Primary myelofibrosis	1 (0.1)
Recurrent sacrococcygeal teratoma	1 (0.1)
Recurrent follicular dendritic cell	1 (0.1)
Unknown primary with cervical met (other)	1 (0.1)
Comorbidities	
Hypertension	76 (5.7)
Diabetes mellitus	72 (5.4)
Thyroid disorders	26 (1.9)
Hepatitis C	17 (1.2)
Hepatitis B	8 (0.6)

Attributes of cancer patients using medications with the potential hazard of QT protraction and TdP

**Figure 1:**
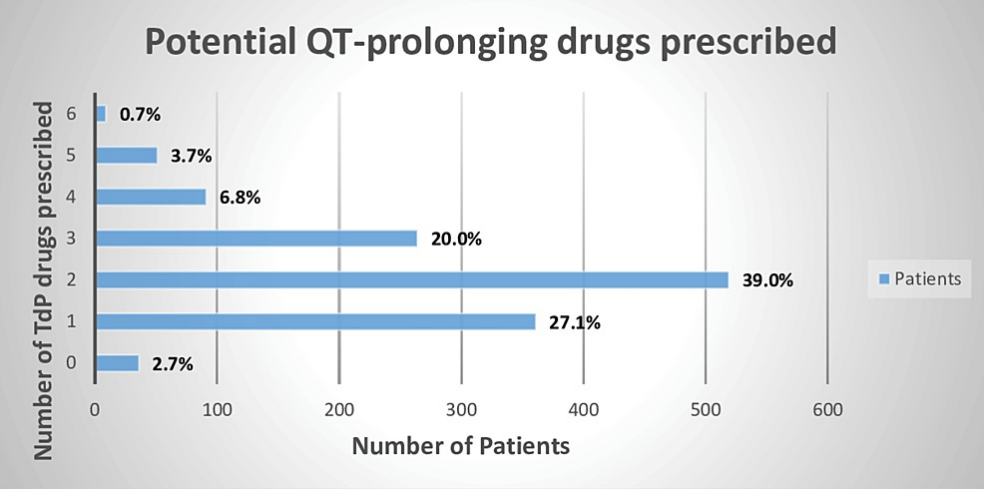
Prevalence of prescription of the potential QT-prolonging drugs The percentage was determined using the total number of patients i.e., 1331 The AzCERT/CredibleMeds QT drugs list was used to assess TdP risk (2nd January 2022) TdP: torsades de pointes; AzCERT: Arizona Center for Education and Research on Therapeutics

**Figure 2:**
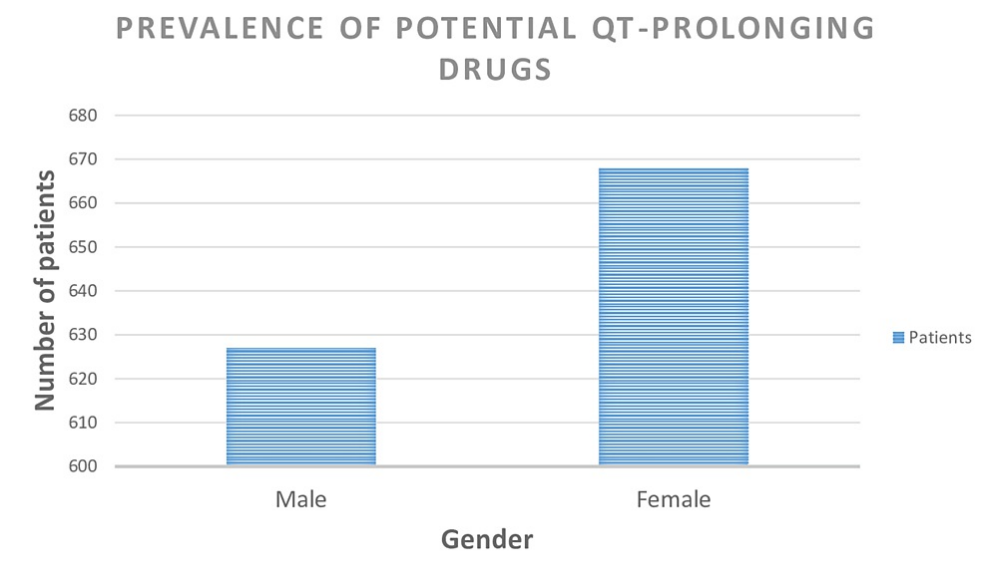
Gender-wise prevalence of prescription of potential QT-prolonging drugs

Among the 1331 cancer patients included in the study, 519 (39.0%) patients were prescribed two drugs that could potentially contribute to TdP risk, while 361 (27.1%) were prescribed one drug with potential TdP risk. Additionally, 264 (19.8%) patients were prescribed three drugs with potential TdP hazards (Figure 1). A total of 2864 potential QT-prolonging drugs were identified in 97.3% of patients. Female patients exhibited a higher prevalence of potential QT-prolonging medication administration compared to male cancer patients, with rates of 668 (50.1%) and 627 (47.1%), respectively (Figure 2).

Prevalence of the prescription of potential QT-interval-prolonging drugs, their therapeutic classes, and TdP risk stratifications

Cancer patients were commonly prescribed medications known to carry the risk of QT prolongation, including antiemetics n=1238 (93.0%), proton pump inhibitors (PPIs) n=632 (47.5%), anticancer drugs n=315 (23.7%), anti-nausea agents n=190 (14.3%), antipsychotic drugs n=159 (11.9%), and antimicrobials n=148 (11.1%), among others, according to the AzCERT/CredibleMeds lists. Among the 54 potential QT-prolonging drugs, ondansetron n=1100 (82.6%), pantoprazole n=616 (46.2%), domperidone n=190 (14.2%), olanzapine n=155 (11.6%), tramadol n=104 (7.8%), and capecitabine n=91 (6.8%) were most commonly used. The six most frequently prescribed anticancer agents with potential QT-prolonging and torsadogenic actions were capecitabine n=91 (6.8%), oxaliplatin n=67 (5.0%), imatinib n= 61 (4.6%), bortezomib n=22 (1.6%), 5-fluorouracil n=21 (1.5%), and bendamustine n=21 (1.5%) (Table 2).

**Table 2:** Prevalence of drugs prescribed that protract the QT interval, their therapeutic classifications, and TdP risk stratifications AzCERT: Arizona Center for Education and Research on Therapeutics; TdP: torsades de pointes; ATC: anatomic therapeutic chemical ^a^Percentage was determined using the total number of patients i.e., 1331 ^b^The AzCERT/CredibleMeds QT drugs list were used to assess TdP risk (2nd January 2022)

Scheme of prevalence/classification	Frequency patients: n(%)^a^
Overall prevalence of prescribed drugs that extend the QT interval	1295 (97.3)

Therapeutic class	TdP risk^b^	QT-interval-prolonging drug (ATC code)	Patients: n (%)^a^
Antimicrobials (n=148)	Possible risk of TdP (n=75)	Ofloxacin (J01RA09)	73 (5.5)
		Norfloxacin (J01RA13)	1 (0.1)
	Known risk of TdP (n=18)	Ciprofloxacin (J01MA02)	11 (0.8)
		Levofloxacin (J01MA12)	5 (0.3)
		Azithromycin (J01FA10)	2 (0.1)
	Conditional risk of TdP (n=7)	Metronidazole (P01AB01)	6 (0.4)
		Piperacillin/Tazobactam (J01CR05)	1 (0.1)
	Special risk (n=48)	Sulfamethoxazole + Trimethoprim (J01EE01)	48 (3.6)
Anticancer (n=315)	Known risk of TdP (n=70)	Oxaliplatin (L01XA03)	67 (5.0)
		Arsenic Trioxide (L01XX27)	3 (0.2)
	Possible risk of TdP (n=245)	Capecitabine (L01BC06)	91 (6.8)
		Imatinib (L01XX28)	61 (4.6)
		Bortezomib (L01XX32)	22 (1.6)
		Bendamustine (L01AA09)	21 (1.5)
		5-Fluorouracil (L01BC02)	21 (1.5)
		Nilotinib (L01XE08)	10 (0.7)
		Dasatinib (L01XE06)	7 (0.5)
		Tamoxifen (L02BA01)	6 (0.4)
		Epirubicin (L01DB03)	2 (0.1)
		Lapatinib (L01EH01)	2 (0.1)
		Eribulin Mesylate (L01XX41)	1 (0.1)
		Leuprolide (L02AE02)	1 (0.1)
Antidepressant (n=3)	Known risk of TdP (n=1)	Escitalopram (N06AB10)	1 (0.1)
	Conditional risk of TdP (n=2)	Amitriptyline (N06AA09)	2 (0.1)
Antidiarrheal (n=33)	Conditional risk of TdP (n=33)	Loperamide (A07DA03)	33 (2.4)
Antiemetic (n=1238)	Known risk of TdP (n=1100)	Ondansetron (A04AA01)	1100 (82.6)
	Conditional risk of TdP (n=59)	Metoclopramide (A03FA01)	59 (4.4)
	Possible risk of TdP (n=79)	Palonosetron (A04AA05)	79 (5.9)
Antifungal (n=12)	Known risk of TdP (n=6)	Fluconazole (J02AC01)	6 (0.4)
	Conditional risk of TdP (n=6)	Voriconazole (J02AC03)	3 (0.2)
		Posaconazole (J02AC04)	2 (0.1)
		Amphotericin B (J02AA01)	1 (0.07)
Antihistamine (n=1)	Conditional risk of TdP (n=1)	Diphenhydramine (R06AA02)	1 (0.1)
Anti-nausea (n=190)	Known risk of TdP (n=190)	Domperidone (A03FA03)	190 (14.2)
Antipsychotic (n=159)	Possible risk of TdP (n=2)	Promethazine (R06AD02)	2 (0.1)
	Conditional risk of TdP (n=155)	Olanzapine (N05AH03)	155 (11.6)
	Known risk of TdP (n=2)	Levosulpiride (N05AL07)	1 (0.1)
		Chlorpromazine (N05AA01)	1 (0.1)
Anti-Tussive (n=6)	Possible risk of TdP (n=6)	Dextromethorphan (N07XX59)	6 (0.4)
Diuretic (n=7)	Conditional risk of TdP (n=4)	Furosemide (C03CA01)	3 (0.2)
		Torsemide (C03CA04)	1 (0.1)
	Possible risk of TdP (n=3)	Hydrochlorothiazide (C03AA03)	3 (0.2)
Proton pump inhibitor (n=632)	Conditional risk of TdP (n=632)	Pantoprazole (A02BC02)	616 (46.2)
		Lansoprazole (A02BC03)	12 (0.9)
		Omeprazole (A02BC01)	4 (0.3)
Analgesic (n=104)	Possible risk of TdP (n=104)	Tramadol (N02AX02)	104 (7.8)
Adrenergic (n=13)	Special Risk (n=12)	Adrenaline (B02BC09)	5 (0.3)
		Salbutamol (R03AC02)	4 (0.3)
		Phenylephrine (S01GA55)	2 (0.1)
		Formoterol (R03AC13)	1 (0.1)
	Conditional risk of TdP (n=1)	Solifenacin (G04BD08)	1 (0.1)
Antiepileptic (n=4)	Possible risk of TdP (n=4)	Levetiracetam (N03AX14)	4 (0.3)

QT-interval-prolonging potential drug-drug interactions (QT-DDIs): prevalence and mechanism

A total of 934 patients received at least two QT-interval-prolonging drugs. Online DDI checkers (drugs.com, Medscape, Epocrates, and Lexicomp) were used to identify 1212 potential QT-DDIs with 102 drug-drug interaction pairs. Among these, 771 (63.6%) QT-DDIs were attributable to pharmacodynamic mechanisms of action, 397 (31.9%) could be explained by pharmacokinetic mechanisms, and 44 (3.6%) were due to unknown mechanisms (Table 3).

**Table 3:** Prevalence of potential drug-drug interactions that extend the QT interval (QT-DDIs) stratified on the basis of gender, age, number of potential QT-DDIs per patient, and mechanisms ^a^Percentage was determined using the total number of patients i.e., 1331 QT-DDI: QT-prolonging drug-drug interactions

Type of prevalence	Patients (n)	%a
Overall prevalence	466	35.0
Gender wise		
Male	266	19.9
Female	200	15.0
QT-DDI per patient		
1-2	299	22.5
3-4	88	6.6
>4	79	5.9
Age-wise prevalence		
≤20	32	2.4
21-40	107	8.0
41-50	107	8.0
51-60	115	8.7
>60	105	7.9
Mechanism of interactions		
Pharmacodynamic	771	63.6
Pharmacokinetic	397	31.9
Unknown	44	3.6

Table 4 shows the summary of QT-drug-drug interactions (QT-DDI) along with their AzCERT/CredibleMeds TdP risk stratification and their therapeutic class or categories. The most frequent potential QT-DDIs identified were domperidone + ondansetron n=122 (10.1%), ondansetron + olanzapine n=114 (9.4%), ondansetron + tramadol n=88 (7.3%), capecitabine + ondansetron n=84 (6.9%), and domperidone + olanzapine n=74 (6.1%).

**Table 4:** Top 20 QT-drug-drug interactions (QT-DDIs) with TdP risks as well as their therapeutic classes AzCERT: Arizona Center for Education and Research on Therapeutics; TdP: torsades de pointes; QT-DDIs: QT-prolonging drug-drug interactions; KR: known risk of TdP; PR: possible risk of TdP; CR: conditional risk of TdP; SR: special risk of TdP ^a^The AzCERT/CredibleMeds QT drugs list were used to assess TdP risk (2nd January 2022) ^b^Percentage computed in total number of QT-DDIs i.e.,1212

QT-DDI (along with TdP risk)^a^	Therapeutic class			Frequency of QT-DDIs: n (%)^b^
	Drug 1	Drug 2		
Domperidone(KR) + Ondansetron(KR)	Antinausea		Antiemetic	122 (10.1)
Ondansetron(KR) + Olanzapine(CR)	Antiemetic		Antipsychotic	114 (9.4)
Ondansetron(KR) + Tramadol(PR)	Antiemetic		Opioid	88 (7.3)
Capecitabine(PR) + Ondansetron(KR)	Anticancer		Antiemetic	84 (6.9)
Domperidone(KR) + Olanzapine(CR)	Antinausea		Antipsychotic	74 (6.1)
Domperidone(KR) + Ofloxacin(PR)	Antinausea		Antimicrobial	67 (5.5)
Ondansetron(KR) + Oxaliplatin(KR)	Antiemetic		Antineoplastic	62 (5.1)
Ofloxacin(PR) + Ondansetron(KR)	Antimicrobial		Antiemetic	59 (4.9)
Metoclopramide(CR) + Ondansetron(KR)	Antiemetic		Antiemetic	57 (4.7)
Olanzapine(CR) + Palonosetron(PR)	Antipsychotic		Antiemetic	42 (3.5)
Sulfamethoxazole/Trimethoprim(SR) + Ondansetron(KR)	Antimicrobial		Antiemetic	36 (3.0)
Loperamide(CR) + Ondansetron(KR)	Antidiarrheal		Antiemetic	32 (2.6)
Capecitabine(PR) + Domperidone(KR)	Anticancer		Antinausea	29 (2.4)
Domperidone(KR) + Oxaliplatin(KR)	Antinausea		Antineoplastic	27 (2.2)
Ondansetron(KR) + Palonosetron(PR)	Antiemetic		Antiemetic	25 (2.1)
Ofloxacin(PR) + Oxaliplatin(KR)	Antimicrobial		Antineoplastic	24 (2.0)
Ofloxacin(PR) + Palonosetron(PR)	Antimicrobial		Antiemetic	24 (2.0)
Ofloxacin(PR) + Olanzapine(CR)	Antimicrobial		Antipsychotic	20 (1.7)
5-Fluorouracil(PR) + Ondansetron(KR)	Anticancer		Antiemetic	17 (1.4)
Capecitabine(PR) + Olanzapine(CR)	Anticancer		Antipsychotic	16 (1.3)

In univariate logistic regression analysis (Table 5), a notably significant association of QT-DDIs was observed for ≤5 drugs (OR=0.02; 95% CI=0.01-0.48; p<0.001), with six to seven prescribed medications (OR=0.05; 95% CI=0.03-0.07; p<0.001), with eight to nine prescribed medications (OR=0.15; 95% CI=0.11-0.21; p<0.001), and ≥10 prescribed medications (OR=13.9; 95% CI=10.6-18.3; p<0.001) for overall prescribed drugs. QT-DDIs were significantly associated with the use of ≥2 torsadogenic drugs, for two TdP-inducing drugs (OR=0.2; 95% CI=0.1-0.3; p<0.001), and ≥3 TdP-inducing drugs (OR=117.0; 95% CI=76.3-179.4; p<0.001). Multivariate logistic regression analysis for QT-DDIs showed significant association with breast cancer (OR=0.3; 95% CI=0.2-0.6; p<0.001), gastrointestinal cancer (OR=1.9; 95% CI=1.0-3.7; p=0.033), genitourinary carcinoma (OR=2.6; 95% CI=1.2-5.8; p=0.015), ovarian cancer (OR=4.8; 95% CI=1.6-14; p=0.003), non-Hodgkin lymphoma (OR=0.2; 95% CI=0.07-0.5; p=0.001), and other carcinomas (OR=1.5; 95% CI=1.1-2.2; p=0.012).

**Table 5:** Data from logistic univariate & multivariate regression analysis for risk factors p-value ≤0.05 considered as significant (*) OR: odds ratio; CI: confidence interval; TdP: torsades de pointes; GI: gastrointestinal cancer; CML: chronic myelogenous leukemia; AML: acute lymphoblastic leukemia; NHL: Non-Hodgkin lymphoma; T2DM: type 2 diabetes mellitus

Variables	Univariate analysis		Multivariate analysis	
	OR (95% CI)	p-value	OR (95% CI)	p-value
Gender	-	-	1.1 (0.7-1.6)	0.6
Male	1.7 (1.4-2.2)	<0.001^*^	-	-
Female	0.5 (0.4-0.7)	<0.001^*^	-	-
Age categories	-	-	1.0 (0.9-1.0)	0.2
≤30	0.7 (0.5-1.0)	0.1	-	-
31-40	0.8 (0.5-1.1)	0.2	-	-
41-50	0.8 (0.6-1.1)	0.3	-	-
>50	1.2 (0.9-1.5)	0.1	-	-
Overall prescribed drugs	-	-	1.0 (0.1-14.8)	0.9
≤5	0.02 (0.01-0.48)	<0.001^*^	-	-
6-7	0.05 (0.03-0.07)	<0.001^*^	-	-
8-9	0.15 (0.11-.021)	<0.001^*^	-	-
≥10	13.9 (10.6-18.3)	<0.001^*^	-	-
Diagnosis				
Breast cancer	0.2 (0.1-0.3)	<0.001^*^	0.3 (0.2-0.6)	<0.001^*^
Head and neck cancer	0.7 (0.4-1.0)	0.1	0.8 (0.5-1.4)	0.6
GI cancer	2.6 (1.6-4.3)	<0.001^*^	1.9 (1.0-3.7)	0.03^*^
Lung cancer	1.2 (0.7-2.1)	0.3	1.0 (0.5-1.9)	0.9
CML	0.2 (0.1-0.4)	<0.001^*^	1.5 (0.5-1.9)	0.3
Genitourinary carcinoma	2.9 (1.5-5.4)	0.001^*^	2.6 (1.2-5.8)	0.01^*^
ALL	0.4 (0.2-0.8)	0.014^*^	0.7 (0.3-1.6)	0.4
Gynecological carcinoma	0.3 (0.1-0.7)	0.007^*^	0.5 (0.2-1.2)	0.1
Ovarian cancer	8.1 (3.4-19.0)	<0.001^*^	4.8 (1.6-14.0)	0.003^*^
NHL	0.4 (0.2-0.8)	0.024^*^	0.2 (0.1-0.5)	0.001^*^
Other carcinomas	1.4 (1.0-2.0)	0.025^*^	1.5 (1.1-2.2)	0.012^*^
Anticancer drugs	-	-	1.1 (0.1-17.0)	0.9
≤2	0.6 (0.4-0.8)	0.006^*^	-	-
>2	1.5 (1.1-2.2)	0.006^*^	-	-
Adjuvant drugs	-	-	1.5 (0.1-21.8)	0.7
≤3	0.02 (0.01-0.04)	<0.001^*^	-	-
4-5	0.03 (0.02-0.04)	<0.001^*^	-	-
6-8	0.1 (0.1-0.2)	<0.001^*^	-	-
>8	14.9 (11.2-20.0)	<0.001^*^	-	-
TdP Drugs	-	-		
1	Omitted	NA	-	-
2	0.2 (0.1-0.3)	<0.001^*^	-	-
≥3	117.0 (76.3-179.4)	<0.001^*^	-	-
Comorbidities	-	-	0.9 (0.6-1.2)	0.5
Hypertension	0.9 (0.5-1.5)	0.7	-	-
T2DM	1.1 (0.6-1.9)	0.6	-	-
Thyroid disorders	0.8 (0.3-1.9)	0.6	-	-
Hepatitis C	0.8 (0.2-2.4)	0.7	-	-
Hepatitis B	0.6 (0.1-3.3)	0.6	-	-
Other comorbidities	1.3 (0.7-2.4)	0.3	-	-

## Discussion

The present study has identified a high prevalence of potential QT-prolonging DDIs (QT-DDIs) in 1295 (97.3%) cancer patients taking anticancer chemotherapy and/or adjunctive medications. In our study, the patients with breast cancer, head and neck cancer, gastrointestinal cancer, and lung cancer happen to be at an elevated risk of TdP owing to frequent use of high-risk QT interval prolonging medications and QT-DDIs involving drugs from AzCERT list 1 (known risk of TdP). Proper scrutiny must be accorded to the monitoring of the effects of these drugs and QT-DDIs in high-risk cancer patients. Polypharmacy and co-morbidities were a major concern in our cancer patients, which may be the reason for the increased frequency of potential QT-DDIs. The most frequent offending drugs were ondansetron, pantoprazole, domperidone, olanzapine, tramadol, and capecitabine. These are documented as being liable for QT-DDIs. The leading antineoplastic agents with potential QT-prolonging and torsadogenic effects most frequently prescribed in our study were capecitabine, oxaliplatin, imatinib, bortezomib, 5-fluorouracil, and bendamustine.

Potential QT-DDIs involving domperidone (KR) ± ondansetron (KR)

Domperidone is known to induce QT prolongation, leading to TdP and sudden cardiac death (SCD) [15-18]. A meta-analysis of six studies noted that domperidone use is strikingly associated with ventricular arrhythmia or cardiac arrest [18]. An epidemiological study carried out in the Netherlands evaluated the use of non-cardiac heart rate-corrected QT(QTc) interval-protracting agents and the hazard of SCD. In a sub-analysis, it was determined that domperidone elevated the risk of SCD by about fourfold [19].

Ondansetron is utilized for postoperative nausea and vomiting and following the administration of emetogenic antineoplastics. TdP evolved in patients subsequent to ondansetron infusions [20] or even after a single intravenous dose of 4mg [21]. Patients had normal baseline QT intervals with no prior cardiac aberrations, but they possessed risk attributes for drug-induced QT prolongation. The pharmacokinetic differences between oral and intravenous ondansetron administration provide a biologically tenable mechanistic rationalization [22]. Another retrospective chart review documented ventricular arrhythmias in some patients who received 0.07-0.15 mg/kg/dose of ondansetron. The patients had critical major cardiac diagnoses like congenital conduction defects, congenital heart anomalies, cardiac tumors, post-heart transplant status, or cardiomyopathy awaiting heart transplant [23]. Globally, regulatory bodies have conveyed concern over the proclivity of 5-HT3 receptor blockers to disturb cardiac conduction. The propensity for QT prolongation with consequent evolution of polymorphic ventricular arrhythmia, like TdP, is the primary apprehension. However, the resting QT interval protraction is a relatively weak foreteller of drug-induced TdP. In vivo, transmural dispersion of repolarization appears to be a stronger teratogenic predictor [24]. Prolongation most frequently arises due to either blockade of potassium-mediated repolarization or type-specific inhibition of cardiac fast sodium channels [25]. For ondansetron, blockade of the human ether-a-go-go-related gene-encoded potassium channel has been implicated as the principal mechanism [25-28]. The US FDA presently advocates abstaining from the use of ondansetron in subjects with congenital LQTS owing to the hazard of TdP. The US FDA endorses uninterrupted cardiac monitoring for subjects receiving ondansetron in concert with electrolyte perturbations, congestive heart failure, or bradyarrhythmia [29].

The clinical use of a combination of domperidone and ondansetron is quite frequent in oncologic practice. For patients who are administered ondansetron in addition to other QT-prolonging agents like domperidone, there may be an escalated hazard of clinically significant QT interval prolongation and subsequent TdP [30,31].

The tendency of ondansetron to impede domperidone biotransformation was investigated in vitro [32]. Ondansetron is biotransformed by CYP3A4 and also by CYP2D6 [33,34]. Additionally, ondansetron has been documented to inhibit CYP3A-mediated metabolic reactions [35,36]. The in vitro experiments were executed in pooled human liver microsomes over the concentration ranges of domperidone and ondansetron that are reached in human plasma at clinical doses. The rate of generation of the main metabolite of domperidone (5-hydroxy domperidone) was probed during the experiment. The study did not provide any evidence that ondansetron impeded the biotransformation of domperidone. These in vitro results presage that the combination of ondansetron and domperidone should be safe at clinical doses from a pharmacokinetic point of view [37].

Potential QT-DDIs involving ondansetron (KR) ± olanzapine (CR)

Practically, second-generation antipsychotics like olanzapine are infrequently related to marked QTc protraction as compared to first-generation agents. In a study utilizing the General Practice Research Database, subjects who were administered olanzapine had a significantly elevated risk of cardiac mortality in comparison with psychiatric patients not receiving antipsychotics [38]. Conditional risk for TdP is associated with olanzapine (with other QTc-prolonging medications and/or decreased potassium/magnesium levels) [39,40]. Olanzapine contains the same warning for QTc prolongation in line with other antipsychotics [41].

Another study examined the dose-related effect olanzapine has on the QTc interval in 21 patients with stable schizophrenia who were administered a starting dose of oral olanzapine (7.1 ± 3.2 mg) for at least two months and then titrated to an escalated dose (18.1 ± 7.5 mg). The QTc interval was significantly prolonged from 396.0 ± 23.8 ms to 404.1 ± 19.0 ms (p=0.031) when patients were administered a higher dose of olanzapine. However, there were no patients who exceeded the upper bound of normal [42].

Long QT associated with a high dose of olanzapine has been documented in the literature [43,44]. For patients who receive ondansetron in addition to other QT-prolonging agents like olanzapine, there may be an escalated hazard of clinically significant QT prolongation and subsequent TdP [30].

Potential QT-DDIs involving ondansetron (KR) ± tramadol (PR)

Tramadol is a synthetic opioid agonist, structurally analogous to codeine and morphine, frequently administered for the control of postoperative, dental, cancer, and musculoskeletal moderate pain, or co-administered with nonsteroidal anti-inflammatory drugs for the control of severe pain.

In patients, tramadol-evoked QTc interval protraction has been observed in good correlation with plasma drug levels [45]. Additionally, a review dwelled on the action of opioids on QTc protraction and their arrhythmogenic proclivity, proposing tramadol as an intermediate-risk drug for the inducing of long QT intervals and TdP at high doses [46]. One study contradicted this evidence, explaining that tramadol does not evoke a clinically pertinent QTc interval extension in healthy subjects with doses up to 600 mg/day [47]. However, it is advocated that patients taking high doses of tramadol should be scrupulously monitored, especially following intravenous administration [48]. There are reports that ondansetron impedes the analgesic efficacy of tramadol. A single 4-mg dose of ondansetron administered at the induction of anesthesia did not reduce the 24-hour incidence of postoperative nausea and vomiting (PONV) [49]. Another study determined that ondansetron decreased the overall analgesic efficacy of tramadol, possibly by inhibiting spinal 5-HT3 receptors [50].

Potential QT-DDIs involving ondansetron (KR) ± capecitabine (PR)

Capecitabine is an orally administered fluoropyrimidine carbamate that functions as an antimetabolite and is biotransformed in vivo to 5-fluorouracil (5-FU). The drug undergoes intestinal absorption with a bioavailability of 80%. It is biotransformed into the pharmacodynamically active substrate 5-FU. It is extensively utilized for the treatment of various malignancies, with colorectal cancer and breast cancer being the most common varieties. Cardiac side effects of capecitabine remain an important problem and resemble those of 5-FU, as capecitabine is biotransformed in vivo into 5-FU. Cardiotoxicity due to 5-FU is the second most common reason for chemotherapy-related cardiac toxicity after anthracyclines [51].

QTc intervals have been noted to increase following capecitabine treatment. More of the patients with anomalous echocardiogram (ECHO) findings prior to capecitabine administration had new-onset cardiovascular problems; more patients with a history of thoracic irradiation had ECG anomalies following treatment; more breast cancer patients had extended QTc intervals; and among the patients with breast cancer, more of those who received trastuzumab treatment had protracted QTc intervals [52,53].

Patients with cardiovascular disease can receive capecitabine, but such patients must be monitored continuously due to the escalated hazard of adverse cardiac side effects. When capecitabine-evoked adverse cardiac side effects manifest, they are expressed as chest pain, arrhythmia, and myocardial infarction, and the outcome is death in 11.3% of patients. When capecitabine-induced adverse cardiac side effects manifest, fluoropyrimidines are preferred (5-FU or raltitrexed could be utilized instead). Since the use of raltitrexed is not commonplace, 5-FU is generally utilized, given as a bolus, and is combined with nitrates or calcium channel blockers. Whereas nitrates are superior in the treatment of myocardial ischemia, the addition of calcium channel blockers is more efficient in averting recurrences [54].

Capecitabine cardiotoxicity is common and often expressed with physical effort. Oncologists, cardiologists, and general practitioners should be mindful of this hazard. Proactive cardiotoxicity monitoring with ECG (concurrently with echocardiogram and/or stress testing in suspected cases) during treatment is advocated [55].

Potential QT-DDIs involving domperidone (KR) ± olanzapine (CR)

Domperidone, ondansetron, and olanzapine may extend the QT interval. The therapeutic utilization of combinations of these medications is very frequent in oncological practice. A study established that QTc protraction is a problem with olanzapine monotherapy and, when combined with domperidone and ondansetron, needs to be examined further [30]. Concurrent administration of several known QT-prolonging medications will be expected to exert an additive effect on cardiac repolarization and hence escalate the proarrhythmic hazard [56].

Potential QT-DDIs involving domperidone (KR) ± ofloxacin (PR)

Domperidone can extend the QT interval (QTc), and life-threatening arrhythmias such as TdP have been reported with its use [57-59].

Cases of TdP have been documented to occur after taking ofloxacin [60-63] as well as levofloxacin (the levo isomer of ofloxacin) [64]. The proarrhythmic risk is not the same for all fluoroquinolones and varies with the administered quantum of the agent. Moxifloxacin is known to exert the greatest impact with regard to QT protraction. Gemifloxacin, levofloxacin, ofloxacin, and ciprofloxacin exert less proarrhythmogenic action. Of these, ciprofloxacin is known to be linked with the least hazard of QT prolongation and the lowest proclivity for TdP.

The overall risk of TdP is minuscule with the administration of fluoroquinolones. Clinicians can minimize the hazard of TdP by abstaining from writing prescriptions for multiple medications documented to cause QT interval prolongation in high-risk outpatients. As such, Katritsis and Camm [65] advocated that ECG surveillance at the start of quinolone therapy is mandatory only if there are underlying factors that predispose the subject to TdP or in those patients receiving multiple medications that could prolong the QT interval [63].

Special caution must be exercised in cases of long QT syndrome or patients with conditions facilitating QT prolongation. In the presence of congestive heart failure, bradycardia, hypokalemia, or hypomagnesemia and the use of drugs that extend the QTc interval independently, such as Class Ia and Class III antiarrhythmic drugs, ciprofloxacin will be the preferred agent, probably with ECG surveillance when starting therapy. The other fluoroquinolone members of the group can also be utilized in such scenarios, but with ECG and/or Holter surveillance during therapy [65].

Potential QT-DDIs involving ondansetron (KR) ± oxaliplatin (KR)

Oxaliplatin is a platinum-based anticancer drug that is extensively utilized in chemotherapy for multiple cancer varieties due to its effectiveness and tractable toxicity attributes. Oxaliplatin may lead to LQTS as it can modify sodium channel kinetics [66]. The detrimental cardiac adverse effects of oxaliplatin may be underestimated because it is extensively co-administered with various antineoplastic agents with documented cardiac toxicity, like 5-FU. Physicians should be mindful of LQTS and TdP as infrequent side effects of oxaliplatin, and hypokalemia and hypomagnesemia must be corrected prior to the utilization of oxaliplatin. The QT interval must be tightly scrutinized regularly before and following the administration of oxaliplatin so that TdP can be avoided. Especially, knowledge of these infrequent side effects of oxaliplatin is critical to reducing risks in susceptible cancer patients with predisposing attributes like electrolytic perturbations, anorexia, and concomitant medications [67-70].

Simultaneous utilization of oxaliplatin and ondansetron escalates the hazards of QT/QTc protraction and TdP. Concurrent use is to be avoided if feasible, particularly in subjects with additional risk factors for TdP. Clinicians must consider taking steps to bring down the hazard for QT/QTc interval protraction and TdP by adopting steps like electrolyte surveillance and repletion and ECG monitoring if concurrent use is imperative. One must be careful not to exceed 16 mg of IV ondansetron in a single dose; the extent of QT prolongation associated with ondansetron markedly increases if this dose is exceeded.

Potential QT-DDIs involving ondansetron (KR) ± ofloxacin (PR)

Simultaneous utilization of ondansetron and ofloxacin escalates the hazards of QT/QTc protraction and TdP. Concurrent use is to be avoided if feasible, particularly in subjects with additional risk factors for TdP. Clinicians must consider taking steps to bring down the hazard for QT/QTc interval protraction and TdP by adopting steps like electrolyte surveillance and repletion and ECG monitoring if concurrent use is imperative.

Potential QT-DDIs involving ondansetron (KR) ± metoclopramide (CR)

Instances of arrhythmia have been documented in the scientific literature with the administration of metoclopramide manifesting as cardiac arrest, bradycardia with subsequent complete heart block, QTc protraction, TdP, supra-ventricular tachycardia, as well as circulatory collapse [71-80]. Proposed underlying or predisposing attributes include prior cardiovascular maladies, atrial fibrillation, autonomous dysfunction, hyperbilirubinemia, halothane anesthesia, and the existence of a pericardial drainage tube. The route and the rate of drug administration may also play a role in instances where an intravenous route is utilized [79].

Possible etiologies for the cardiac arrhythmias could be the blockade of presynaptic auto-receptors and more powerful catecholamine release; promotion of cholinergic neurotransmission; and finally, 5-HT3 receptor blockade or 5-HT4 receptor agonism, which may result in QT protraction [81]. Another study examined the impact of utilizing ondansetron and metoclopramide for prophylaxis of postoperative nausea and vomiting. It was noted that in 100 healthy subjects, 10 mg of metoclopramide extended the QT interval from 13.2 ± 1 to 19 ± 1 msec [82].

Genetic diversity exerts an impact on drug pharmacokinetics and, hence, modulates the drug’s action and the proclivity to engender side effects. A study facilitated determining patients who are likely to reap benefits from metoclopramide and those who will develop side effects [83]. Clinical efficacy was observed in patients with elevated body mass index (BMI), and side effects manifested more in females, non-diabetics, and patients with normal gastric emptying. Pharmacogenomic scrutiny unraveled that the most compelling predictors of effectiveness were polymorphisms in the KCNH2 and ADRA1D genes, whereas polymorphisms in the CYP2D6 and serotonin 5-HT4 receptor HTR4 genes were linked with escalated side effects. This study failed to detect any causal relationship between ABCB1 allelic variants and the effectiveness of metoclopramide or the development of its side effects. Specific apportioning of DRD3 allelic variants proposes a mechanistic role for DRD3 in the development of adverse effects following metoclopramide administration. In the patient group with no side effects, there was a higher representation of a minor allele (rs7625282: G). Additionally, in the same group, heterozygous genotypes were overrepresented, suggesting that the minor allele might have a defensive action [83].

Genetic testing has appreciable relevance to evaluating the therapeutic activities of metoclopramide and reducing the side effects. However, more extensive studies are imperative to assess and corroborate the interindividual pharmacogenetic diversities before it becomes a frequent practice [84].

Potential QT-DDIs involving olanzapine (CR) ± palonosetron (PR)

Chemotherapy-induced nausea and vomiting (CINV) is a troublesome and routine adverse event related to cancer treatment. The individual patient risk of CINV is correlatable to the type of chemotherapy utilized and distinct patient attributes. Agents like cisplatin and dacarbazine have profound emetogenic capability with emesis in almost all patients, while carboplatin, anthracyclines, and cyclophosphamide (<1500 mg/m2) are deemed to be moderately emetogenic, with greater than 30% of patients encountering emesis. Etoposide, gemcitabine, and mitoxantrone have low emetogenic capability, with emesis occurring in 10-30% of subjects. Age of less than 50 years, female gender, history of low prior chronic alcohol intake, history of motion sickness, and emesis during pregnancy are notable risk factors for CINV. The second-generation 5-HT3 receptor antagonist palonosetron was licensed for the deterrence of CINV depending upon several phase II-III trials in 2003, and contemporary studies propose that it has prolonged efficacy in containing delayed CINV compared to the first-generation 5-HT3 receptor blockers [85]. Palonosetron may evoke QTc protraction [86] (especially during the early general anesthesia period when administered with sevoflurane) [87]. However, there have been no electrocardiographic or dose-response relationships, including QTc extension of any clinical apprehension, for palonosetron up to a 2.25 mg intravenous dose, a ninefold safety leeway [88,89].

Potential QT-DDIs involving ondansetron (KR) ± sulfamethoxazole/trimethoprim (SR)

Cotrimoxazole may influence myocardial repolarization. High doses escalate the likelihood that repolarization will be influenced [90]. Individual instances of cotrimoxazole-related TdP have been documented [91,92].

Potential QT-DDIs involving ondansetron (KR) ± loperamide (CR)

At therapeutic dosing, cardiac conduction aberrations and dysrhythmia have not been documented with loperamide administration; thus, it is speculated that the cardiac effects noted are a dose-related anomaly cropping up only at supra-therapeutic dosing. Very recently, several instances of cardiac dysrhythmias and protraction of the QRS and QTc intervals have been attributed to loperamide misuse [93-98].

QT interval extending DDIs with anticancer agents

Patients with cancer possess numerous risk factors for developing arrhythmias [99-101]. An overall prevalence of 1295 (97.3%) of QT-DDIs in this study is greater in comparison with other studies executed in oncology settings. A Pakistani study reported QT-prolonging drug use in 92.6% of the patients, involving a total of 28 distinct QT-prolonging medications [6]. Many drugs marginally extend the QT interval, but in some patients, this protraction can be excessive and provoke the morphologically typical polymorphic ventricular arrhythmia (VA) TdP. QT-protracting DDIs could lead to fatal outcomes like TdP. Symptoms correlatable to TdP could be syncope and SCD if the arrhythmia is prolonged or degenerates into ventricular fibrillation. More recently, it has been documented that a few antineoplastic agents, like ibrutinib (a Bruton’s tyrosine kinase inhibitor), can also evoke fatal VA without prolonging the QT interval [101]. The primary objective of utilizing drug combinations in cancer therapeutics is directed towards facilitating exposure of neoplastic cells to optimal drug concentrations to achieve maximum inhibition of cancer cell multiplication. However, polypharmacy is a primary hazard for DDIs stemming from QT protraction [6]. Concerning QT-interval-protracting DDIs, there is a paucity of data regarding the risks of concurrent usage of QT-protracting medications and how these DDIs should be handled. DDIs inducing QT interval extension must be preferably avoided in patients.

In our present study, the total number of DDIs is positively correlated with the total number of medications and the number of extant comorbidities (Table 5). This signifies that as the number of drugs increases, there is an escalated risk of DDIs as well as adverse effects in cancer patients taking antineoplastic medications. This observation is consistent with a few other studies reported in the medical literature, where an increase in the number of drugs prescribed has been linked with increments in DDIs [102-104]. The hazard factors for polypharmacy in patients with cancer are the number of health problems, inpatient admissions, and prescriptions to manage adverse drug reactions with adjunctive medications. The repercussions of polypharmacy in oncology are adverse drug reactions, DDIs, incremental hazards of morbidity, and mortality [105-107].

It is possible that every physician and pharmacist is unable to recollect and comprehend all pertinent DDIs and hence cannot implement corrective actions correspondingly. They may be more conversant with the medications employed in their specialty but not with drugs prescribed in other specialties. Commonplace launches of new drugs and licensing of new indications for marketed drugs make the identification of instances of DDIs more problematic for healthcare professionals. To deal with that, various DDI screening programs or databases have been devised and utilized as clinical decision support tools. A tool that clinicians depend upon to review patients’ medication sheets for DDIs is computerized DDI packages. DDI-checking/screening algorithms are extensively engaged to determine potentially detrimental drug interactions in inpatient and outpatient scenarios. What is crucial is that these software packages differ in accuracy and the information within interaction monographs. Past studies have reported discrepancies (lack of consistency in the inclusion and grading of major drug interactions) across different DDI-checking databases/programs [108-110]. Information relevant to the interacting drugs and epidemiological study resource accessibility and reliability is vital for each interaction monograph. Clinical pertinence of DDIs assessed with DDI software is an exclusive apprehension as the software is unable to account for the patient’s attributes, dosing scheme, and safeguards instituted by the clinicians. Hence, DDIs identified by the electronic databases could be over-detected in comparison to the clinician’s evaluation. Studies have documented a high level of inconsistency between the frequency of DDIs detected by electronic software and the frequency of clinically pertinent DDIs evaluated by a clinician. DDI-checking databases and programs may invoke evidence from a study without a control group for interaction to discern confounding factors. Out of the three online DDI-checking programs utilized by us for this study, Drugs.com included references to establish DDI scientific evidence. The discrepancy between electronic software databases in the severity rating of determined DDIs can be possibly due to the inconsistency of evidence and disparate criteria for the classification of DDI severity by diverse software [111]. A judicious approach would be to check more than one DDI-checking database or program and compare their outputs to glean optimum sensitivity and specificity. Additionally, the perceptive reasoning of the clinician is of crucial importance to ascertain relevance from trivial interactions.

The strengths of this study were that it was executed prospectively and that the wisdom of concerned clinicians in relation to the potential QT-DDIs was deliberated upon and taken into cognizance regarding their clinical pertinence to a certain extent.

Recommendations for attenuating the hazards of QT-DDIs in oncology clinical settings

In the context of cancer patients, it is important to take steps to reduce the hazards stemming from QT-DDIs. Multiple safeguards have been proposed, and clinicians have been advised to heed them when prescribing medications that have the capability to evoke QT-DDIs.

Thorough scrutiny of concurrent medication prescriptions is essential. If concomitant medications are necessary, mandatory biological and clinical monitoring should be established. Some drugs may require dosage adjustments or discontinuation. If discontinuation is not feasible, the offending drug can be replaced with an alternative with a lower risk of causing QT-DDIs [112].

Secondly, the collaborative and educational role of clinical pharmacologists/pharmacists is the cornerstone of evidence-grounded monitoring of the medications advised for each patient and, hence, more efficient identification and thwarting of QT-DDIs. Our study highlights the commonplace prescription of QT-interval-protracting drugs in concert with ECG underutilization in oncology patients. Being a low-priced, non-invasive, and generally accessible test, ECG may be readily incorporated into the surveillance of patients for toxicities in routine clinical practice [113].

Thirdly, patients afflicted with cancer (or their family members as well as caregivers) have a role to play in educating themselves and forging self-awareness regarding the hazards/warning signs, and symptoms of QT-DDIs (stemming out of administered/self-administered mechanisms).

Fourthly, clinicians have the entitlement to utilize several online DDI-checking software packages to investigate the possible QT-DDIs of prescribed drugs and institute necessary adjustments to preempt them accordingly. Physicians must educate and alert patients about the possible hazards of interactions evoked by medications and the potential perils of self-medication.

Fifthly, there is a pressing requirement to harmonize and standardize the multitude of online DDI-checking software packages and numerous DDI-related resources and make them highly user-friendly, accessible, comprehensive, and algorithmic in approach (with inbuilt risk-stratification and alerting options).

Finally, the importance of artificial intelligence- and machine learning-based automated schemes such as computerized prescribing order entry and knowledge/non-knowledge-based clinical decision support tool workflows for facilitating decision-making by clinicians (medication prescribers) cannot be overemphasized, though their procurement costs could be exorbitant and thus, their availability is presently confined to a handful of centers [114-116].

However, the absence of baseline ECG measurements represents a significant limitation of our study. While our prospective design enabled real-time identification of potential drug-drug interactions (DDIs) and subsequent clinical alerts to healthcare providers, the lack of baseline ECG data hampers our ability to directly correlate reported QT prolongations due to DDIs with clinical outcomes. Baseline ECG measurements are crucial for establishing individual QT intervals and determining any pre-existing QT prolongation before the initiation of potentially interacting medications. Another limitation is the confined scope of data collection from a solitary institution. Consequently, the study's findings may not be universally applicable to different geographical contexts, given its conduct within a single, large academic tertiary care teaching hospital in northern India.

## Conclusions

Cancer patients are at high risk of QT-DDIs ascribable to polypharmacy. So, the treating oncologist must carefully scrutinize and cut down on the list of medications before administering antineoplastic chemotherapy. The treating physician may utilize the most updated AzCERT QT drug lists in concert with online DDI-checking software to pinpoint the potential QT-DDIs and can monitor and modify the medications judiciously. An integrative and algorithmic surveillance mechanism with the inclusion of oncologists, cardiologists, general practitioners, and clinical pharmacologists/pharmacists is imperative to optimize drug therapy and minimize detrimental medication effects in patients afflicted with cancer.
